# BioSearch: a semantic search engine for Bio2RDF

**DOI:** 10.1093/database/bax059

**Published:** 2017-08-08

**Authors:** Wei Hu, Honglei Qiu, Jiacheng Huang, Michel Dumontier

**Affiliations:** 1 State Key Laboratory for Novel Software Technology, Nanjing University, China; 2 Institute of Data Science, Maastricht University, The Netherlands

## Abstract

Biomedical data are growing at an incredible pace and require substantial expertise to organize data in a manner that makes them easily findable, accessible, interoperable and reusable. Massive effort has been devoted to using Semantic Web standards and technologies to create a network of Linked Data for the life sciences, among others. However, while these data are accessible through programmatic means, effective user interfaces for non-experts to SPARQL endpoints are few and far between. Contributing to user frustrations is that data are not necessarily described using common vocabularies, thereby making it difficult to aggregate results, especially when distributed across multiple SPARQL endpoints. We propose BioSearch — a semantic search engine that uses ontologies to enhance federated query construction and organize search results. BioSearch also features a simplified query interface that allows users to optionally filter their keywords according to classes, properties and datasets. User evaluation demonstrated that BioSearch is more effective and usable than two state of the art search and browsing solutions.

**Database URL:** http://ws.nju.edu.cn/biosearch/

## Introduction

Over the last few years, there has been a groundswell of institutional support for using Semantic Web technologies to make life science data easily findable, accessible, interoperable and reusable. Major biomedical data providers, including EBI (1), NCBI (https://www.ncbi.nlm.nih.gov/), NLM (http://id.nlm.nih.gov/mesh) and SIB (2), followed the linked data principles and provided life science Linked Data to public access. Bio2RDF (3), Neurocommons (4), Chem2Bio2RDF (5), Protein Data Bank Japan (6) and W3C HCLS LODD (http://www.w3.org/wiki/HCLSIG/LODD) also conducted efforts towards establishing Linked Data over hundreds of life science datasets. Data in these sources are generally available in RDF formats (e.g. RDF/XML, Turtle and JSON-LD), as well as being queryable using SPARQL, a standardized query language for RDF (http://www.w3.org/TR/rdf-sparql-query/). While powerful, SPARQL may be challenging to formulate for users without technical training. Composing a good query also needs high familiarity with the dataset (7, 8). Therefore, developing effective methods to query these data is key for increased adoption and reuse across the life sciences and health care communities as well as the Semantic Web community.

To date, *keyword search* is the most popular paradigm to retrieve information on the Web. It is also widely adopted by applications to the life sciences, such as GoWeb (9) and Drug Encyclopedia (10). However, keywords are often ambiguous and could have multiple meanings. For example, given a keyword ‘Alzheimer’, the user intent can be searching the information for this disease or for some drugs to treat it. To address this problem, *semantic search* was proposed to improve search accuracy by understanding user intent and search context (11). It particularly suits to investigative search (as compared to navigational search), where a user gathers a number of data which together will provide the desired information.

Another main obstacle in querying life science Linked Data lies in its heterogeneity. While the use of Semantic Web standards such as RDF and OWL aids in providing a uniform representation across data structures, at least at the syntactic level, it is now the diversity of vocabularies that makes it more challenging to get all the data of interest (e.g. an issue of completeness). Ontologies, which are formal representations of knowledge in a domain, can be used to unify the different data vocabularies by matching classes, properties and instances with one another. So, *ontology-based query answering* (a.k.a. ontology-based data access) aims to realize the semantic interoperability between applications using different biomedical datasets (12–14).

In this paper, we introduce *BioSearch*, a semantic search engine that leverages semantic search and ontology-based query answering over a wide range of life science Linked Data, obtained from Bio2RDF. The main contributions of this paper are summarized as follows:

- We define an Effective Accessible Semantic quEry interface (EASE), which accepts not only plain keywords but also three types of semantic tags to specify the class, property and dataset constrains. EASE can support a set of frequently-used SPARQL queries in Bio2RDF and be easily learned without technical training.
- We leverage the Semanticscience Integrated Ontology [SIO, (15)] for the mediating ontology and use hierarchical relations between ontology classes to conduct query expansion (13). By using ontology matching to match classes and properties in SIO with each target dataset, we propose an ontology-based query answering method to rewrite a query against SIO to a query against the dataset semantically.
- We design a fine-grained presentation to show the life science Linked Data. Search results can be filtered using various facets and entity descriptions are clustered in groups. Additionally, users can follow links to traverse across different datasets.
- We developed an online system for BioSearch, and conducted user-involved experiments to compare it with two widely-used SPARQL endpoint solutions on four different groups of tasks. Our experimental results demonstrated that BioSearch gained significantly better effectiveness and usability (in average +16.5% and +30.5%, respectively) than the next best system for searching and browsing the life science Linked Data.

The remainder of the paper is structured as follows. In Section 2, we describe the system architecture of BioSearch and the statistics of currently involved datasets. In Section 3, we present our methods for semantic query interface, ontology matching, mapping-based query answering and entity browsing and filtering. Evaluation is reported in Section 4, and related work is discussed in Section 5. Finally, we conclude the paper with future work in Section 6.

## Overview of biosearch

An *RDF dataset* is comprised of a set of *RDF triples* that are often published, maintained, and aggregated by a single provider. An *RDF triple* consists of three components: the subject, which is a URI or a blank node; the predicate, which is a URI; and the object, which is a URI, a literal or a blank node. An *ontology* may include *classes*, *properties*, *instances* and their descriptions. Classes and properties together are often referred to as *terms*, while instances are referred to as *entities*. A SPARQL query contains a set of triple patterns matching a subgraph of RDF data, where a triple pattern contains variables to match the RDF triples. The query result contains the terms and entities substituting for the variables in the matched subgraph.

**Figure 1.**
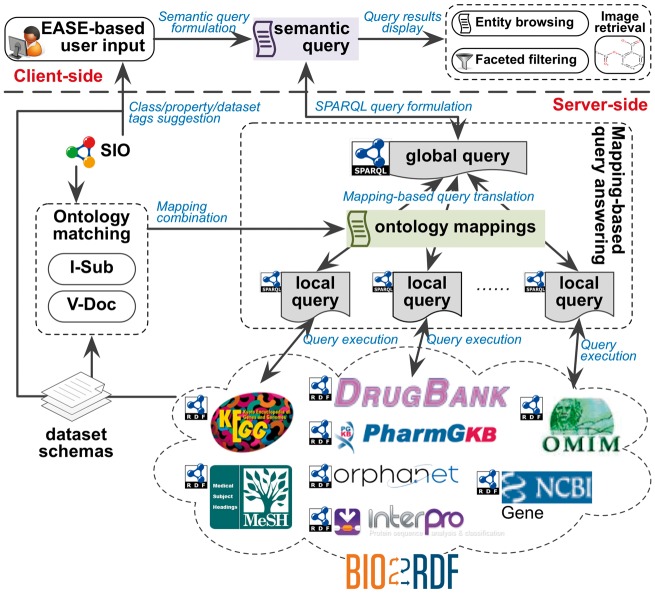
BioSearch architecture.

The architecture of BioSearch is depicted in Figure 1, which follows the widely-used Client-Server paradigm (more specifically, the Browser-Server paradigm) providing the system with high bandwidth, processing and storage on a large amount of data. Furthermore, the Global-as-View [GAV, (16)] solution is chosen for data integration, because it is very efficient for query processing and the used datasets are not frequently updated. This flexible architecture enables us to continue adding more life science datasets without paying much extra effort.

We select eight Bio2RDF datasets, Drugbank (http://www.drugbank.ca/), InterPro (http://www.ebi.ac.uk/interpro/), KEGG (http://www.genome.jp/kegg/), MeSH (http://www.nlm.nih.gov/mesh/), NCBI Gene (http://www.ncbi.nlm.nih.gov/gene/), OMIM (http://www.omim.org/), Orphanet (http://www.orpha.net/), PharmGKD (http://www.pharmgkb.org/), based on their popularity and coverage (Table 1). We briefly describe four important components in BioSearch as follows:

**Table 1.** Statistics of involved Bio2RDF Release 3 datasets and SIO

Names	Entities	Classes	Prop.	RDF triples
DrugBank	316 950	91	105	3 672 531
InterPro	176 579	30	41	2 323 345
KEGG	6 533 307	63	141	50 197 150
MeSH	305 401	7	61	7 323 864
NCBI Gene	185 330 642	34	70	1 966 214 397
OMIM	1 013 389	34	101	8 750 774
Orphanet	28 871	19	38	377 947
PharmGKB	25 325 504	50	88	278 049 209
SIO	0	1471	235	11 120

The statistics were queried on December 10, 2015.

- EASE-based user interface accepts user inputs containing plain keywords as well as class, property and dataset constrains and formulates the semantic queries based on SIO and the language of EASE. These semantic queries are passed to the server side for searching Bio2RDF datasets.
- *Ontology matching* component leverages two linguistic matchers V-Doc (19) and I-Sub (20) to match classes and properties in SIO with each Bio2RDF dataset schema. The offline-computed mappings are used by the mapping-based query answering component for SPARQL query rewriting.
- *Mapping-based query answering* component accepts a semantic query from the client side and formulates it to a ‘global’ SPARQL query w.r.t. SIO. Based on the mappings between SIO and Bio2RDF datasets, this SPARQL query is rewritten as a set of ‘local’ SPARQL queries and executed on the target Bio2RDF datasets.
- *Entity browsing and faceted filtering* component organizes query results and display them to users. Faceted filters are dynamically constructed based on the query results. Additionally, users can follow links to traverse across different datasets.

**Figure 2.**
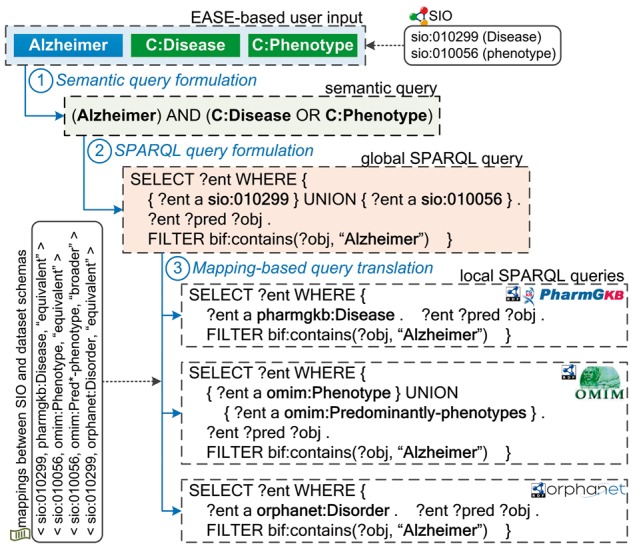
A running example.

A typical workflow of query answering in BioSearch is shown in Figure 2. Assume that a user first submits an EASE-based query to BioSearch, which contains a plain keyword **Alzheimer** and two semantic tags (class constraints) **C:Disease** and **C:Phenotype**. Next, this query is formulated to a semantic query according to SIO and the definition of EASE (Step 1) and then rewritten to a ‘global’ SPARQL query against SIO (Step 2). Based on the pre-computed mappings between SIO and Bio2RDF dataset schemas, the ‘global’ query is semantically translated to a set of ‘local’ SPARQL queries against some Bio2RDF datasets like PharmGKB (Step 3). Finally, the query results are collected and returned to the user for further browsing and filtering.

## Methods

### EASE: Effective Accessible Semantic quEry interface

One of our main goals is to find a balance between simple keyword search and complex SPARQL query. Using five million SPARQL queries against Bio2RDF between May 12, 2013 and September 28, 2014, Buil-Aranda *et al.* (17) observed that almost 50% of the queries just consist of a single triple pattern plus one filter (e.g. containing some text), suggesting that most users are specifically looking for matches using a search phrase. The second most popular query involves a **Describe**, which yields the set of information relevant to a specific entity. The authors concluded that the general usage of SPARQL in Bio2RDF is very basic and the users do not know the datasets well, which motivated us to design a simple, but effective accessible semantic query interface called EASE. Based on the previous analysis (17), EASE can directly support >50% of the SPARQL queries from our logs, which is admittedly more than we anticipated when we set out to design a simpler interface. Moreover, ontology-based integration with SIO means that there are fewer classes and properties for users to worry about, and it directly offers users a simpler way to query the information in Bio2RDF — without knowing the intricacies of namespaces and mappings that would otherwise be required. The ease of use was demonstrated by our user study (Section 4), which revealed that the users thought that BioSearch (featuring EASE) was substantially easier to use than either keyword queries or the controlled natural language of Sparklis (Q3 in Table 7, scores: 3.87, 2.47 and 2.27, respectively).

At the end of the day, we firmly believe that a multiplicity of interfaces will improve adoption of Semantic Web technologies. We think that BioSearch will be well used by some subset of our users, e.g. biomedical users who are non-experts in SPARQL. Even for users familiar with SPARQL, they do not always require high expressiveness but sometimes want to find information quickly, thus BioSearch can be a good option. In fact, our current Bio2RDF portal, openlifedata.org, features three interfaces: BioSearch, Virtuoso SPARQL Query Editor, and an API. In future work, we hope to study how these interfaces are naturally being used by our users, but this is beyond the scope of our current study.

The definition of EASE is shown in Table 2, which receives four different categories of keywords. In addition to plain keywords like ‘penicillin’, we define three kinds of semantic tags to constrain the searching scope, where the class tag ‘C:’ limits the *class* or type of entities, the property tag ‘P:’ compels entities to have the specified *property*, and the dataset tag ‘S:’ gives the *source* of entities. The set of class tags are derived from the SIO, the mediating ontology. To better fit the Bio2RDF datasets, we also extend SIO with 54 commonly-used properties from RDF(S) (e.g. **rdfs:label**), OWL (e.g. **owl:sameAs**), DC (e.g. **dc:title**) and FOAF (e.g. **foaf:homepage**).

**Table 2.** Definition of the effective accessible semantic query interface

Keyword types	Regular	Expressions examples
Plain text	^ (?![CPS]:)\w+	penicillin
Class constraint	^ C:\w+	C:Drug
Property constraint	^ P:\w+	P:label
Dataset constraint	^ S:\w+	S:DrugBank

‘\w’ denotes case-insensitive alphanumeric characters.

We use ‘AND’ logic to combine keywords across the four categories while ‘OR’ logic is used for multiple keywords within the same category. For instance, the semantics of the query ‘**alzheimer C:Phenotype C:Disease’** is ‘**(Alzheimer) AND (C:Phenotype OR C:Disease)’**. Using the ‘OR’ logic to combine keywords within the same category may produce many search results containing only one keyword. To avoid the users to spend too much effort to find the expected result, in practice we rank the results that simultaneously contain more keywords within the same category higher, similar to traditional search engines like Google.

**Figure 3.**
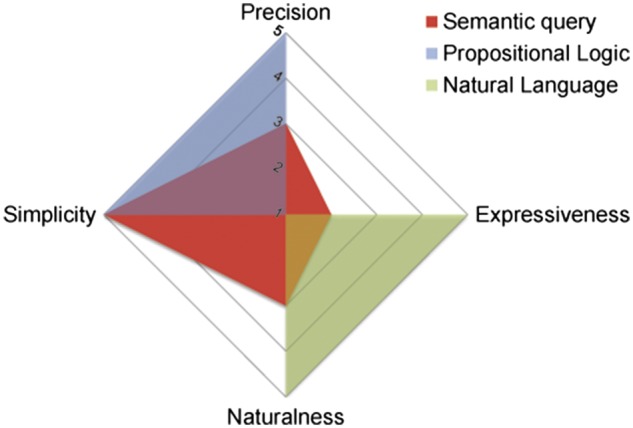
PENS scores for the effective accessible semantic query language.

According to the analysis framework of controlled languages in (18), the scores of EASE are shown in Figure 3. In comparison to the propositional logic and natural language (e.g. English), our semantic query interface is very simple, moderately natural and precise, as well as weakly expressive, which meets our goal to make it easier to search the life science Linked Data for non-technical users in the biomedical domain. Complex logic queries are beyond EASE’s competence, but faceted filtering can partly make up and we would like to extend EASE to support more expressive information needs in the future. Also, iteratively search on previous results are expected.

We employ keyword auto-completion for the users who are not familiar with the classes or properties in the mediating ontology SIO. The query interface shows candidate classes, properties or data sources when a user types in ‘C:’, ‘P:’ or ‘S:’. Additionally, faceted filtering provides the users dataset-specific classes and properties that are not involved in SIO. The details of faceted filtering are described in Section 3.4.

### Ontology matching

Different terminologies are often used in different datasets. Therefore, answering queries across datasets requires that either the data are integrated to a common terminology (e.g. warehouse), or that a mediator translates a query against target sources. In our work, we adopt the latter, in which we establish mappings between Bio2RDF classes and properties to the SIO ontology, and execute queries by translating them to the target datasets via these mappings. We use ontology matching to find corresponding classes and properties between Bio2RDF datasets and SIO.

To match an ontology with SIO, we use two linguistic methods, namely V-Doc (19) and I-Sub (20), which are both from our Falcon-AO ontology matching system (21). We select it due to its stable performance in several years’ OAEI contests (http://oaei.ontologymatching.org/), but other alternative systems, e.g. S-Match (22), can be used for this purpose as well. Note that comparing the performance of different ontology matching systems is beyond the scope of this paper. Structural methods are ignored here due to the weak structures of Bio2RDF schemas.

V-Doc forms a virtual document, denoted by VD(), to represent each term. The novelty of V-Doc is that both local descriptions, denoted by LD() and neighboring information are involved to increase the robustness. For a literal, its local description is a bag of words from its lexical form; while for a term, it is a bag of words extracted from the local name of its URI or other annotations. To incorporate the descriptions of neighbors in a virtual document, we define three neighboring operations to cover different kinds of neighbors: subject neighbors SN(), predicate neighbors PN() and object neighbors ON(). Also, synonyms from MeSH are queried to enrich the documents. The measure to determine whether two terms are similar is the cosine similarity of their virtual documents. Specifically, let $t_{s}$, $t_{b}$ be two terms to be matched, the similarity of them using V-Doc is defined as follows:

(1) $$V-\text{Doc}(t_{s},t_{b})=\frac{VD(t_{s})•VD(t_{b})}{\left|VD(t_{s})\right|\cdot \left|VD(t_{b})\right|},$$

(2) $$\begin{array}{c} VD(t)=LD(t)+\gamma_{s}\sum_{t^{\prime }\in SN(t)}LD(t^{\prime })+\gamma_{p}\sum_{t^{\prime }\in PN(t)}LD(t^{\prime })\\ +\gamma_{o}\sum_{t^{\prime }\in ON(t)}LD(t^{\prime }), \end{array}$$

where $\gamma_{s}$, $\gamma_{p}$, $\gamma_{o}$ are weighting factors in range [0,1], and they are recommended to be 0.5 (19). ‘•’ denotes the dot product of two vectors.

I-Sub is an improved string matcher considering not only the commonalities between the local descriptions of two terms but also their differences. Specifically, let $t_{s}$, $t_{b}$ be two terms to be matched, the similarity of them using I-Sub is defined as follows:

(3) $$\begin{array}{c} I-\text{Sub}(t_{s},t_{b})=\text{Comm}(LD(t_{s}),LD(t_{b}))–\text{Diff}(LD(t_{s}),LD(t_{b}))\\ \;\;\;\;\;\;\;\;\;\;\;\;\;\;\;\;\;\;\;\;\;\;\;\;\;\;\;\;\;\;\;\;\;\;\;\;\;\;\;\;\;\;\;\;\;+Winkler(LD(t_{s}),LD(t_{b})), \end{array}$$

where Comm() and Diff() measure the commonality and difference of the two local descriptions, respectively, and Winkler() is a refinement.

To meet various use scenarios, the overall similarity between two terms are measured as the maximal value of V-Doc and I-Sub (21):

(4) $$\text{Sim}(t_{s},t_{b})=\max\{V-\text{Doc}(t_{s},t_{b}),I-\text{Sub}(t_{s},t_{b})\}.$$

Note that this similarity combination strategy is completely different from the original one used in Falcon-AO. Another difference is that synonyms from MeSH are complemented to resolve the semantic heterogeneity. Furthermore, the threshold for the overall similarity is adjusted to 0.9, in order to achieve a high accuracy.

Table 3 lists the class and property mappings between SIO and eight Bio2RDF dataset schemas. We invited an expert to judge the quality of the class mappings and found that the precision and recall are very good (Table 3). Our approach finds not only simple mappings between string variations (e.g. **sio:010038** (drug) vs. **kegg:Drug**), but also lexically problematic mappings such as **sio:010299** (disease) vs. **orphaned:Disorder**. However, some mappings such as **sio:011125** (molecule) vs. **drugbank:Small-molecule** are missing due to conservative thresholds. One mapping between **sio:000133** (descriptor) and **mesh:Descriptor** was marked as wrong due to **sio:000133** is broader than **mesh:Descriptor**, rather than equivalent. We evaluated the quality of property mappings. A majority of the properties in these mappings for each Bio2RDF dataset are the same as the commonly-used properties (e.g. **rdfs:label**) that we added to SIO. We observed that the derived mappings are all correct. It is worth noting that our architecture supports to use other ontologies as the mediating ontology to filter classes and properties in different datasets. However, most biomedical ontologies currently focus on one specific domain in the life science [e.g. Basic Formal Ontology, (23)] or provide domain-independent classes and properties [e.g. Human Phenotype Ontology, (24)]. Using these ontologies would decrease the coverage of the matched classes and properties.

**Table 3.** Matching results between SIO and Bio2RDF datasets

Names	Class mappings	Property mappings	Precision	Recall
			of class mappings	
DrugBank	9	54	1.00	0.90
InterPro	5	50	1.00	1.00
KEGG	14	52	1.00	1.00
MeSH	8	49	0.88	0.88
NCBI Gene	6	50	1.00	1.00
OMIM	9	52	1.00	1.00
Orphanet	7	40	1.00	1.00
PharmGKB	9	51	1.00	0.82

Table 4 lists the coverage rate of the matched classes and properties against the involved Bio2RDF datasets. In average, about 45% classes and properties are covered by SIO. The coverage on DrugBank is not good, because it contains many classes specific to drugs (e.g. **Half-life**) while SIO does not have them currently. However, the frequently-used classes (e.g. **Drug**, **Disease**, **Gene**) and properties (e.g. **dc:title**, **rdfs:label**) have all been matched. NCBI Gene and PharmGKB also encounter the similar problem. The low coverage would lead to the failure of our mapping-based query rewriting, because a ‘global’ query against SIO cannot be translated to the ‘local’ query against a specific Bio2RDF dataset. We alleviate this shortage by using faceted filtering, which provides the users dataset-specific classes and properties that are not involved in SIO. To clarify, we did not use expert judgement to improve the mappings in our experiments.

**Table 4.** Coverage rate of matched classes and properties against the involved Bio2RDF datasets

Names	Coverage rate of	
	classes	Properties
DrugBank	12.3%	38.0%
InterPro	41.7%	63.3%
KEGG	66.7%	29.1%
MeSH	66.7%	45.8%
NCBI Gene	30.0%	47.6%
OMIM	69.2%	37.4%
Orphanet	43.8%	52.6%
PharmGKB	33.3%	38.6%

### Mapping-based query answering

With equivalence mappings, the queries against SIO are immediately converted to the queries against a specific dataset. A class constraint is translated to a triple pattern 〈?s a c〉, and a property constraint is translated to 〈?s p  ?o〉, where c, p denote the matched class and property in that specific dataset, respectively. Plain keywords are converted to SPARQL filters, and dataset constraints are converted to named graphs. One pattern in each type is combined to constitute a SPARQL query, and all combinations form the federated query. For the dataset-specific classes and properties that cannot find mappings in SIO, the users can use faceted filtering instead, in order to refine the information of interest.

*Example 1.* To help understand the mapping-based query answering, let us recall the running example in Figure 2. Assume that a user wants to search information about ‘Alzheimer’. To make her query more accurate, she specifies the class must be **Disease OR phenotype** (Steps 1-2). This information need is automatically translated to a SPARQL query against SIO (Step 3). Then, this query is rewritten into local Bio2RDF datasets based on the discovered mappings. For example, Orphanet uses **Disorder** instead of **Disease**; OMIM has two disjoint classes **Phenotype** and **Predominantly-phenotypes**. Thereafter, the disease and phenotype information about Alzheimer can be queried from distributed life science datasets.

### Entity browsing and faceted filtering

The returned entities are displayed with highlighted keywords. The external image of each entity (if available) is also retrieved on the fly. To view the details of an entity, because it may have hundreds of properties and values, we arrange the properties by categorizing them into three groups: the metadata group (e.g. properties from RDF(S), OWL or DC), the domain-specific group, and the entity-linking group. Within each group, properties and values are ordered alphabetically. The entity-linking group mainly consists of the x-link relations (e.g. **kegg:x-pharmgkb**), which form a majority of entity links in Bio2RDF (25). These cross-references enable users to easily traverse linked entities across different datasets.

Also, we extract the classes, properties and datasets of the returned entities to construct faceted filters, with which users can further refine search results. Extracted classes and properties are organized as the leaf nodes in a tree structure with the non-leaf nodes being the matched SIO classes and properties, or being the data source names for the unmatched ones, so that the dataset-specific classes and properties would not be ignored. Note that both property grouping, facet organization and term mappings can be configured based on application scenarios.

### Implementation details

**Figure 4.**
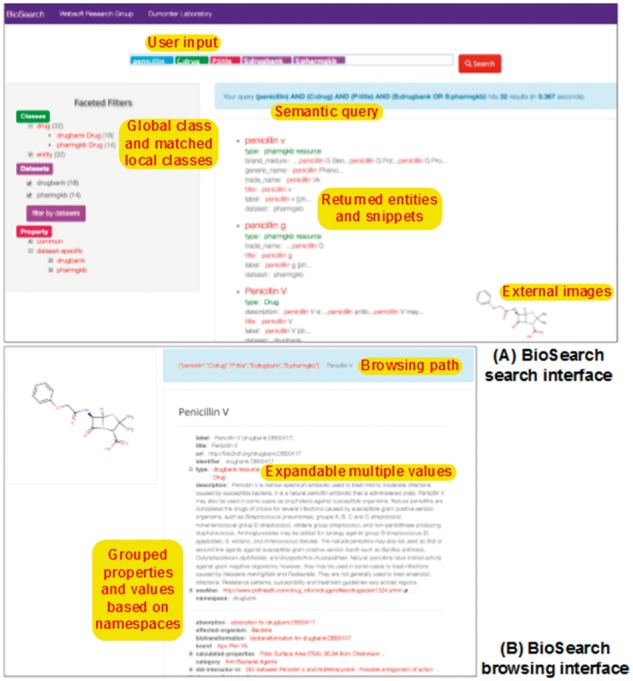
User interface of BioSearch.

We implemented the server side of BioSearch in Java and the browser side in JavaScript. Biosearch is currently deployed on an IBM x3850 M2 server with two Xeon Quad 2.4 GHz CPUs and 16GB memory, using Apache Tomcat as the Web server on CentOS Linux. SPARQL queries are serialized using Apache Jena and submitted to different SPARQL endpoints, typically using OpenLink Virtuoso to store RDF data. The user interfaces of BioSearch are shown in Figure 4, where the drugs about ‘penicillin’ are found in DrugBank and PharmGKB. The current implementation of BioSearch depends on SIO and its mappings with Bio2RDF datasets. With a proper mediating ontology and ontology mappings, we believe that BioSearch can be applied to other application domains without taking too much effort.

## Evaluation

In this section, we report our experiments to evaluate BioSearch in addition to two comparative systems with user-involved tasks. We mainly aim to test the following two hypotheses:

H1. Combining traditional Web search and semantic query is more effective than using one of them in the life science Linked Data.

H2. Conducting ontology-based query answering is more effective than querying isolated datasets in the life science Linked Data.

### Comparative systems

**Figure 5.**
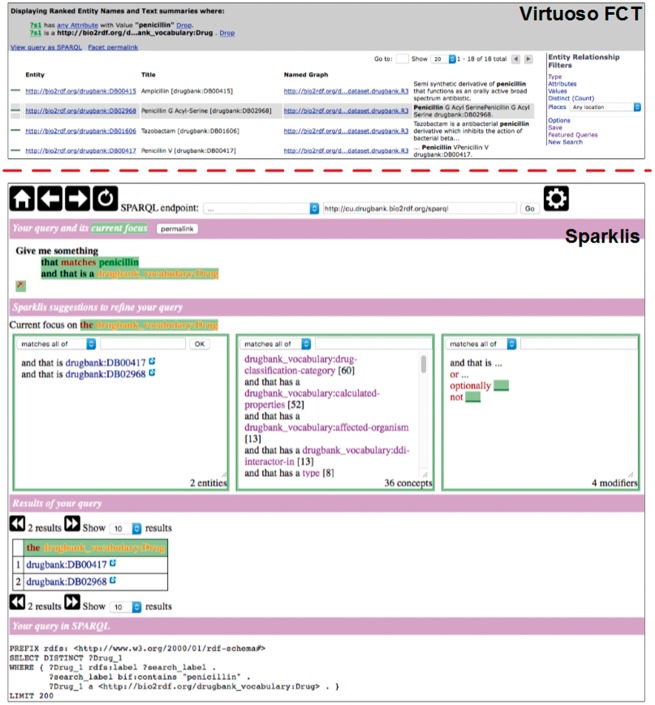
User interfaces of Virtuoso FCT and Sparklis.

We compared BioSearch with two types of widely-used SPARQL endpoint solutions: one follows the classic keyword search paradigm just like Google; the other aims at facilitating SPARQL query construction. In our tests, we chose two state of the art Web applications – the Virtuoso search and faceted browser [VFCT in abbr., (26)] and Sparklis (27) – and deployed them to work with the same datasets as BioSearch. The user interfaces of VFCT and Sparklis are shown in Figure 5, and we briefly introduce them below:

- VFCT is currently the Bio2RDF query engine for full-text search and faceted browsing. For a dataset, it accepts plain-text keywords as input just like Google search engine and helps users refine search results with entity relationship filters (see the top-right corner of Figure 5).
- Sparklis facilitates users to explore a SPARQL endpoint via directing them to the interactive construction of questions and answers. For a SPARQL endpoint, it combines the fine-grained guidance of faceted search, most of SPARQL expressivity, and readability of controlled natural languages.

Alternatively, we may use SPARQL query editors like YASGUI (http://about.yasgui.ort/) or Lodestar (http://www.ebi.ac.uk/fgpt/sw/lodestar/) for our intended comparison. But we left them out of consideration, since this would bring too heavy burdens for non-technical domain users to participate in the experiments. We also omitted existing biomedical search engines like NCBI search (http://www.ncbi.nlm.nih.gov/) or EBI search (http://www.ebi.ac.uk/), because they run on different datasets and schemas, which are difficult for us to compare. The Optique Platform supports federated queries and helps non-IT experts construct complex queries (28). Currently, we are unable to obtain the software and deploy it on Bio2RDF. However, comparing the Optique Platform with BioSearch for the ontology-based query answering would be our important future work.

### Users, tasks and procedure

*Users.* We invited 30 domain users to participate in the evaluation. 10 of them are biologists and physicians (denoted by BP), and they have adequate knowledge of the life sciences but limited knowledge of the Linked Data; another 10 users are graduate students studying the Semantic Web (denoted by SW), but they are unfamiliar with the life sciences; the remaining 10 users are professionals in Biomedical Informatics, who know both areas very well (denoted by Both). The three groups of users with varied backgrounds reflect diversity (29), and we can compare their opinions on the three systems.

*Tasks.* We designed 12 tasks of four categories, listed in Table 5, to search entities or associations within a single dataset or across different datasets in Bio2RDF. The tasks involve some well-known drugs, diseases and genes, and represent common scenarios to use Bio2RDF according to the query log analysis in (17) and our interview on its users from Stanford University who use Bio2RDF frequently. We selected the keywords (e.g. Smallpox) in the tasks by ourselves, but they are independent to EASE, and no property constraint is required to complete the tasks. For equity, we explicitly gave the dataset names (except for three enumeration tasks T4, T5, T6), since VFCT and Sparklis do not support federated queries on multiple datasets at present, and some users (especially from the SW group) may be not very familiar with the datasets.

**Table 5.** Tasks for effectiveness and usability assessment

	Entity search	Association search
Single dataset	T1/T2/T3	T7/T8/T9
Multiple datasets	T4/T5/T6	T10/T11/T12

T1. Find the description of drug ‘Penicillin V’ in DrugBank.

T2. Find the category of disease ‘Smallpox’ in KEGG.

T3. Find the synonym of gene PHF8 in NCBI Gene.

T4. Find the identifiers of gene ‘BRCA1’ in any two datasets.

T5. Find the URIs of drug ‘Norgestrel’ in any two datasets.

T6. Find the entity whose disease is Malaria in any two datasets.

T7. Find the interaction between two drugs ‘Fluoxetine’ and ‘Phenelzine’ in DrugBank.

T8. Find the association between disease ‘Schizophrenia’ and gene ‘DRD2’ in PharmGKB.

T9. Find the association between warfarin and gene APOC1 in PharmGKB.

T10. Find the toxicity of a drug in DrugBank that treats disease ‘Tuberculosis’ in KEGG.

T11. Find the dosage of a drug in DrugBank that treats disease ‘Syphilis’ in KEGG.

T12. Find the indication in DrugBank of a drug that treats disease Type I diabetes in KEGG.

*Procedure.* Before experiment, a tutorial and training exercise (i.e. Example 1) was given for each user to understand the functionality of the three systems. Then, the user conducted a randomly assigned task in each category using BioSearch, VFCT and Sparklis arranged in a balanced order, that is, each system was rotated to different places equally, and was required to finish the task in 10 min. For example, one user was arranged to conduct (T1, T5, T9, T10) on VFCT, (T3, T4, T8, T11) on BioSearch and (T2, T6, T7, T12) on Sparklis. Completion time of each task was recorded.

Once users completed the tasks on all the three systems, they then completed a survey (Table 6) on domain familiarity and task difficulty, a customized questionnaire (Table 7), which we designed to assess information quality, functionality and response time of the systems, and the standard System Usability Scale (SUS) chart (30) which is widely accepted to evaluate the general system usability. Note that, although there is some overlap between our questionnaire and the SUS chart, our questionnaire was specifically customized for this evaluation and more focused on the effectiveness of the systems rather than the usability in SUS. We used a five-point Likert item from 1 for ‘strongly disagree’ to 5 for ‘strongly agree’. We then calculated the average scores and significance in statistics (e.g. repeated measures ANOVA) to compare the effectiveness and usability of the systems. We also evaluated users’ performance by analysing completion recall, precision and time consuming of conducting tasks. Finally, we collected and analysed any additional comments.

**Table 6.** Questions and results for task difficulty and domain familiarity

Questions	Scores: Mean (SD)			LSD post-hoc
	BP	SW	Both	(*P* < 0.01)
Q1. This domain is familiar to me.	2.80	2.19	3.08	Both, BP > SW
	(1.12)	(1.43)	(2.62)	
Q2. These tasks are difficult to me.	2.80	2.79	2.27	SW, BP > Both
	(1.12)	(1.41)	(1.34)	

Task difficulty (*P* < 0.001): T10/T11/T12 > T7/T8/T9, T4/T5/T6 > T1/T2/T3.

**Table 7.** Questions and results for effectiveness assessment

Questions	Scores: Mean (SD)			*F*(2, 87)	LSD post-hoc
	BioSearch	VFCT	Sparklis	(*P*-value)	(*P* < 0.01)
Q1. This system provided me with accurate information.	4.50	3.60	3.20	16.183	BioSearch > VFCT, Sparklis
	(0.63)	(0.97)	(1.06)	(< 0.001)	SW, BP, Both
Q2. This system provided me with abundant information.	3.73	3.63	2.87	6.799	BioSearch, VFCT > Sparklis
	(0.94)	(1.03)	(1.01)	(< 0.005)	SW, BP, Both
Q3. This system helped me easily find information of interest.	3.87	2.47	2.27	24.55	BioSearch > VFCT, Sparklis
	(1.01)	(0.82)	(1.05)	(< 0.001)	Both, SW > BP
Q4. This system helped me clearly understand related information.	3.73	2.70	2.47	13.851	BioSearch > Sparklis, VFCT
	(0.91)	(1.15)	(0.90)	(< 0.001)	Both> BP
Q5. This system rapidly responded to my queries.	3.70	3.90	2.53	14.441	VFCT, BioSearch > Sparklis
	(1.06)	(0.99)	(1.14)	(< 0.001)	SW, Both > BP

### Results and discussion

*Domain familiarity and task difficulty.* Table 6 lists the average scores, including means and standard deviations (SD), of the 10 users in each group. Users with the life science background (i.e. in BP and Both) thought that they are more familiar with this domain and users known both areas felt easier to finish the assigned tasks. One-way ANOVA reveals the significant difference (*P* < 0.005). However, all the users confirmed that they had no difficulty in understanding the English tasks.

Concerning the three tasks in each category, there is no significant difference in statistics between them (*P* > 0.41), which excludes the factor that task assignment would affect user scoring. However, all the users agreed that the association search tasks among multiple datasets are harder and entity search tasks in single dataset is easier (*P* < 0.001).

*Effectiveness.* Table 7 illustrates the result of the questionnaire for effectiveness assessment, where the former two questions focus on information quality while the latter three aim at system functionality and response time. From the table, we find that BioSearch achieved the highest scores on the first four questions. Repeated measures ANOVA indicates the significant difference in statistics among Q1, Q2, Q3 and Q4 (*P* < 0.005), and LSD post-hoc test reveals that BioSearch is consistently better than VFCT and Sparklis. For Q2, Spaklis provided less abundant information (*P* < 0.005). For Q5, BioSearch used slightly more time on responding. Table 8 shows the effectiveness assessment results of different user groups. For Q3, Q4 and Q5, users in BP group gave lower points for all three systems (*P* < 0.001).

**Table 8.** Effectiveness assessment results of different user groups

Questions	User group	Scores: Mean (SD)		
		BioSearch	VFCT	Sparklis
Q1	BI	4.2 (0.60)	4.1 (0.70)	3.1 (0.94)
	SW	4.6 (0.49)	3.4(1.02)	3.7 (0.90)
	Both	4.7 (0.64)	3.3 (0.90)	2.8 (1.08)
Q2	BI	3.3 (0.64)	3.4 (0.66)	3.0 (0.77)
	SW	3.8 (0.98)	3.8 (0.98)	3.1 (1.04)
	Both	4.1 (0.94)	3.7 (1.27)	2.5 (1.02)
Q3	BI	2.8 (0.75)	2.1 (0.70)	1.8 (0.60)
	SW	4.4 (0.49)	2.5 (0.81)	2.3 (1.10)
	Both	4.4 (0.66)	2.8 (0.75)	2.7 (1.10)
Q4	BI	3.0 (0.63)	2.5 (0.92)	2.2 (0.60)
	SW	3.9 (0.70)	2.3 (0.64)	2.6 (0.80)
	Both	4.3 (0.78)	3.3 (1.42)	2.6 (1.11)
Q5	BI	2.7 (0.90)	3.2 (1.08)	2.1 (0.83)
	SW	4.1 (0.70)	4.4 (0.66)	2.9 (1.51)
	Both	4.3 (0.64)	4.1 (0.70)	2.6 (0.66)

*Usability.* Table 9 presents the average SUS scores of the sytems, where BioSearch achieved the highest score and Sparklis obtained the worst. Repeated measures ANOVA demonstrates the significant difference in statistics (*P* < 0.001), and LSD post-hoc test confirms this. Table 10 shows the detailed SUS scores of different user groups. Users in BP group gave lower points for all three systems because they were not such familiar with Semantic Web and ontology. Based on (31), an SUS score above 68 would be considered above average, thus the score of BioSearch (72.28) indicates it usable.

**Table 9.** SUS results for usability assessment

Scores: Mean (SD)			*F*(2, 87)	LSD post-hoc
BioSearch	VFCT	Sparklis	(*P*-value)	(*P* < 0.01)
72.28	50.25	32.25	49.941	BioSearch> VFCT> Sparklis
(17.36)	(16.48)	(12.29)	(< 0.001)

**Table 10.** SUS results of different user groups

User Group	Scores: Mean (SD)		
	BioSearch	VFCT	Sparklis
BI	50.33 (4.35)	44.50 (10.89)	30.50 (10.59)
SW	84.75 (10.27)	51.50 (17.47)	34.50 (12.24)
Both	81.75 (4.75)	54.75 (17.59)	31.75 (12.94)

*User Performance.* Users’ performance on conducting tasks is described in Table 11 using recall, precision and completion time, where BioSearch achieved best recall and precision with least time. Users finished more tasks on BioSearch. Repeated measures ANOVA indicates the significant difference in statistics between BioSearch and Sparklis (*P* < 0.005). For completed tasks, users achieved slightly better precision on BioSearch, and repeated measures ANOVA indicates the difference is significantly different (*P* < 0.005). Users spent less time on BioSearch to complete each task, and repeated measures ANOVA indicates the difference is significantly different (*P* < 0.001). Table 12 shows the detailed performance results of different user groups, where users in BP group got a lower completion recall and spent much more time on tasks because of their unfamiliarity (*P* < 0.001).

**Table 11.** Results for user performance

	Scores: Mean (SD)			LSD post-hoc
	BioSearch	VFCT	Sparklis	(*P* < 0.01)
Recall	0.94	0.89	0.78	BioSearch > Sparklis
	(0.11)	(0.17)	(0.23)	
Precision	0.94	0.90	0.90	BioSearch > VFCT, Sparklis
	(0.11)	(0.18)	(0.13)	
Time (s)	198	239	328	BioSearch < VFCT < Sparklis
	(154)	(167)	(187)

**Table 12.** User performance results of different user groups

	User group	Scores: Mean (SD)		
		BioSearch	VFCT	Sparklis
Recall	BI	0.85 (0.12)	0.78 (0.18)	0.64 (0.23)
	SW	1.00 (0.00)	0.93 (0.16)	0.83 (0.20)
	Both	0.98 (0.08)	0.90 (0.08)	0.98 (0.08)
Precision	BI	0.92 (0.13)	0.93 (0.13)	0.92 (0.13)
	SW	0.95 (0.10)	0.84 (0.20)	0.89 (0.13)
	Both	0.95 (0.10)	0.93 (0.16)	0.90 (0.12)
Time (s)	BI	317 (187)	341 (183)	420 (184)
	SW	139 (73)	208 (146)	291 (178)
	Both	136 (94)	169 (111)	272 (160)

*User behavior.* Let us take T10 for example. Using the VFCT solution, a user might first initiate a keyword search ‘tuberculosis’ on the Bio2RDF KEGG database and then select disease **kegg:H00342** from 316 unordered results simply containing this keyword. Using the Type facet, the user could then manually examine the entries for a disease type entry. Selecting this, the user could then find the **kegg:drug** property and follow the link for **kegg:D00144**. The user would then have to use the **dc:title** or the **dc:identifier** as a keyword search on the DrugBank VFCT. Using the title, the user would have to further examine or refine the 11 results to identify **drugbank:DB00339**.

On Sparklis, the user first specifies the KEGG SPARQL endpoint and searches for ‘tuberculosis’ in the entity column. However, a common issue is that the user cannot find any KEGG types in the concept column due to user-configurable application limit to the number of search results. The user must then manually type **kegg:Disease** to filter the desired entity. The steps that follow are then similar to VFCT.

With BioSearch, the user initiates her search by typing the keywords ‘tuberculosis’, ‘C:Disease’ and ‘S:KEGG’. From the short list of results, the user then clicks on the official tuberculosis entry, chooses one drug name (e.g. Pyrazinamide), and can immediately follow the link to a DrugBank entry as specified with **kegg:x-drugbank**. However, the current semantics of EASE does not support the user to directly express the input query with **kegg:x-drugbank**, because it is dataset-specific.

*User comments.* We summarized all the major comments that were made by at least 20% users. 70% of the users appreciated the user-friendly interface of BioSearch, especially the semantic query input and faceted filtering. 33% of the users said that it is clear in semantics to search with BioSearch across multiple data sources based on ontology-based query answering. On VFCT, 27% of the users said that it is also easy to use, however, 50% of the users complained that its result presentation is less than ideal. For instance, VCFT lacks snippets in an accessible format, and the facets are not obvious to the user. 30% of the users thought that Sparklis can make complex queries step by step, but 40% of the users believed that this needs a lot of training before actually using it, especially for the modifier column. 27% of the users commented that a weakness of Sparklis is it can only query a single SPARQL endpoint at a time. Users complained that VFCT and Sparklis require to remember and type in endpoint addresses when switching datasets.

*Discussion.* The above reported scores, user behavior and comments are consistent between different groups of the users, so we discussed comprehensively. These results support our two hypotheses.

To H1, BioSearch leveraged keyword search and semantic query for finding information more accurately and efficiently. Only using keyword search like VFCT failed to interpret user intent precisely, while constructing semantic queries with Sparklis was difficult for users without technical training.

To H2, BioSearch conducted ontology-based query answering to automatically retrieve information from distributed datasets, which not only realized the federated SPARQL queries but also coped with the heterogeneity issue with ontology matching. Neither VFCT nor Sparklis supported this presently. Users need to switch datasets manually and repeat typing in very similar queries to retrieve the federated search results.

However, there are some issues that have not been well covered in our evaluation. First, the motivation of EASE is to support biomedical users who are not familiar with SPARQL or the schemas of Bio2RDF datasets, but its current expressiveness is relatively limited. The users sometimes need to divide their information needs into several steps of querying, browsing and filtering (e.g. as explained by T10). Enriching the expressiveness of EASE or using another mediating ontology has not been conducted in the evaluation yet.

Second, the ontology-based query answering retrieves information from distributed Bio2RDF datasets, but our main focus is to resolve the heterogeneity between different dataset schemas. It cannot support complex federated queries compared with existing federated SPARQL query engines, e.g. (28). In future work, we plan to make an in-depth comparison on their capability and efficiency of query answering, to better assess the benefits of different components in BioSearch.

## Related work

Semantic Web search engines such as Swoogle (32), Sindice (33), Falcons (34) have been developed to index an increasing amount of RDF data. They accept keyword queries and display relevant results similar to conventional search engines, and are intended to be general and do not cover the life science domain well (9). Different from these IR-based search engines, semantic search (11) aims to provide more accurate results, which transforms keyword queries to formal SPARQL queries. User interaction is often considered (27), which may be difficult for users without technical skills.

In the life science and health care domains, GoWeb (9) combines keyword search with ontologies and text mining to navigate large results sets and facilitate query answering. BioGateway (35) provides a single-entry point to query ontologies in the OBO foundry, GO annotation files, SWISS-PROT protein set, NCBI taxonomy and several in-house ontologies through SPARQL. Bioqueries (36) is a wiki-based portal to aid domain users in developing SPARQL queries to access biological linked data. FedViz (37) is a step towards interactively formulating federated SPARQL queries using classes and properties visually presented per dataset. GoPubMed (38) exploring PubMed with the GO to filter results. QuerioDALI (39) provides a question-answering interface to search against biomedical data sources. These systems cannot query distributed datasets with heterogeneous vocabularies and different data types. More generally, ontology-based data access is a paradigm for accessing data sources through an ontology that acts as an integrated view of the data, and declarative mappings that connect the ontology to the data sources (12–14, 28, 40). In BioSearch, we specifically used SIO as the mediating ontology and conducted automated ontology matching for query translation.

To handle heterogeneity, Linked Biomedical Dataspace (41) integrated data from multiple sources by a mediating model named CanCO for cancer chemoprevention drug discovery. Drug Encyclopedia (10) proposed the data mart to represent the Linked Data and enables physicians to faceted search and browse clinically relevant information about drugs. Disease ontology cancer project (42) matched cancer terms from different dataset and provides a website for keyword search. Compared with them, BioSearch uses an automated ontology matching method and supports semantic search over distributed datasets.

## Conclusion

In this paper, we described a semantic search engine BioSearch for the life science Linked Data, using Bio2RDF as our main source. BioSearch leverages semantic search and ontology-based query answering for information retrieval from distributed datasets. The proposed semantic query interface is effective to support major use scenarios, and the ontology matching method resolves the heterogeneity between different dataset schemas. Also, entity browsing, filtering and traversing can be all easily conducted. Our evaluation involving both non-technical and technical domain users showed that BioSearch is more effective and usable than two existing systems. In the future, we will continuously integrate new datasets and functions. We will also extend the query interface to support more expressive queries. Additionally, we will try to combine our ontology-based query answering with other federated SPARQL query engines.
